# Ribosome profiling in mouse hippocampus: plasticity-induced regulation and bidirectional control by TSC2 and FMRP

**DOI:** 10.1186/s13229-020-00384-9

**Published:** 2020-10-14

**Authors:** Annie Hien, Gemma Molinaro, Botao Liu, Kimberly M. Huber, Joel D. Richter

**Affiliations:** 1 Program in Molecular Medicine, University of Massachusetts Medical School, Worcester, MA 01605 USA; 2 Medical Scientist Training Program, University of Massachusetts Medical School, Worcester, MA 01655 USA; 3 Department of Neuroscience, University of Texas Southwestern Medical Center, Dallas, TX 75390 USA

## Abstract

**Background:**

Mutations in *TSC2* are the most common cause of tuberous sclerosis (TSC), a disorder with a high incidence of autism and intellectual disability. TSC2 regulates mRNA translation required for group 1 metabotropic glutamate receptor-dependent synaptic long-term depression (mGluR-LTD) and behavior, but the identity of mRNAs responsive to mGluR-LTD signaling is largely unknown.

**Methods:**

We utilized *Tsc2*^+/−^ mice as a mouse model of TSC and prepared hippocampal slices from these animals. We induced mGluR-LTD synaptic plasticity in slices and processed the samples for RNA-seq and ribosome profiling to identify differentially expressed genes in *Tsc2*^+/−^ and following mGluR-LTD synaptic plasticity.

**Results:**

Ribosome profiling reveals that in *Tsc2*^+/−^ mouse hippocampal slices, the expression of several mRNAs was dysregulated: terminal oligopyrimidine (TOP)-containing mRNAs decreased, while FMRP-binding targets increased. Remarkably, we observed the opposite changes of FMRP binding targets in *Fmr1*^*−/y*^ hippocampi. In wild-type hippocampus, induction of mGluR-LTD caused rapid changes in the steady-state levels of hundreds of mRNAs, many of which are FMRP targets. Moreover, mGluR-LTD failed to promote phosphorylation of eukaryotic elongation factor 2 (eEF2) in TSC mice, and chemically mimicking phospho-eEF2 with low cycloheximide enhances mGluR-LTD in TSC mice.

**Conclusion:**

These results suggest a molecular basis for bidirectional regulation of synaptic plasticity and behavior by TSC2 and FMRP. Our study also suggests that altered mGluR-regulated translation elongation contributes to impaired synaptic plasticity in *Tsc2*^+/−^ mice.

## Background

Tuberous sclerosis (TSC) is an autosomal dominant disorder characterized by benign tumor growth in multiple organs and neuropsychiatric symptoms. Individuals with TSC have an increased incidence of seizures (~ 90%), intellectual disability (~ 50%), and autism (~ 50%) [1]. Disrupted neuronal circuitry likely underlies many neuropathologies in TSC because individuals with an anatomically normal brain can still present substantial developmental delay, intellectual disability, and autism. More direct evidence for impaired neuronal connectivity is derived from mouse models of the disorder, which display hyperexcitability, aberrant synaptic plasticity, and altered dendritic spine morphology [2, 3]. TSC is caused by loss-of-function mutations in *TSC1* or *TSC2*; mutations in *TSC2* are more common and are responsible for the most severe symptoms. The TSC1/2 proteins heterodimerize to form the tuberous sclerosis complex (TSC), a GTPase-activating protein that inhibits Ras homology enriched in brain (Rheb) [4, 5]. Rheb activates the mechanistic target of rapamycin (mTOR), a kinase that forms two biochemically distinct complexes: mTORC1 and mTORC2 [6]. mTORC1 regulates mRNA translation primarily via activation of the kinase p70S6K to phosphorylate rpS6 and 4E-BP [7–9]. The less-characterized mTORC2 plays a role in actin cytoskeletal reorganization [6]. Complete loss of *TSC1* or *TSC2* leads to excessive mTOR activity, and mutations in other components of the mTOR pathway are also linked to autism in humans such as *PTEN*, *RHEB*, and *MTOR* [10, 11]. Thus, the mTOR pathway forms a highly connected signaling network linked to autism. Although pharmacological options are available for the treatment for seizures in individuals with TSC, there currently is no effective pharmacological treatment for the neuropsychiatric symptoms of intellectual disability and autism.

The mTOR pathway integrates signaling from various inputs to regulate synaptic plasticity and higher cognitive function. One example is metabotropic glutamate receptor-mediated long-term depression (mGluR-LTD), a form of synaptic plasticity that requires de novo protein synthesis [12]. The mGluR1/5 agonist dihydroxyphenylglycine (DHPG) stimulates group 1 metabotropic glutamate receptors to activate the PI3K/AKT/mTOR pathway, leading to translation of largely unidentified mRNAs required for mGluR-LTD [13, 14]. Multiple mouse models of autism, such as TSC and Fragile X Syndrome, have altered hippocampal mGluR-dependent synaptic plasticity [2, 15–17]. Although experiments with rapamycin and inhibitors of protein synthesis suggest that excessive mTORC1-dependent translation drives the altered synaptic plasticity phenotype in TSC mice, other findings suggest a more complex picture. For example, *Tsc2*^+/−^ mice have reduced hippocampal protein synthesis rates instead of the predicted excessive protein synthesis from mTOR hyperactivation [2]. In addition, a recent study challenged the role of mTORC1 in hippocampal mGluR-LTD, arguing that mTORC2 is the major regulator of this form of synaptic plasticity [18, 19] but see [13, 20]. MGluR1/5 signaling can also regulate translation factors to suppress global protein synthesis, such as phosphorylation of eukaryotic initiation factor 2 alpha and eukaryotic elongation factor 2 (eEF2), which are both necessary for mGluR-LTD [18, 21]. Although mGluR1/5 stimulation is known to promote translation of specific mRNAs [22], how translation is regulated in a transcriptome-wide manner by mGluR1/5 in either the normal or TSC brain is unknown.

To study the role of TSC2 in synaptic plasticity, we generated a germline *Tsc2*^+/−^ mouse and performed ribosome profiling, an unbiased whole transcriptome method that determines the number and positions of ribosomes on all mRNAs and thus serves as a proxy for protein synthesis [23]. Together with RNA-seq, ribosome profiling in wild-type and *Tsc2*^+/−^ hippocampal slices under basal conditions and after induction of mGluR-LTD allows us to define posttranscriptional changes in gene expression in response to synapse activation [12]. We chose the *Tsc2*^+/−^ mouse model, because we strove to capture translational dysregulation that arises from *TSC2* haploinsufficiency as in humans with TSC. This mouse model displays excessive mTOR signaling, a well-characterized deficient mGluR synaptic plasticity, and alterations in social interactions and hippocampal-dependent learning tasks that are reversed by rapamycin treatment [2, 24–26]. Using ribosome profiling, we identified changes in the *Tsc2*^+/−^ hippocampus as well as following mGluR-LTD signaling. We also observed that mGluR1/5 stimulation induces a rapid increase in mRNA levels, and targets of Fragile X Mental Retardation Protein (FMRP), an RNA-binding protein whose loss causes Fragile X Syndrome, are enriched in this group. mGluR stimulation also promotes phosphorylation of eukaryotic elongation factor (eEF2), which is deficient in *Tsc2*^+/−^ mice. These animals have a reduced mGluR-LTD, and chemically mimicking phospho-eEF2 enhances this form of synaptic plasticity. Our results suggest a novel pathway of translational dysregulation in ribosome translocation (polypeptide elongation) that may contribute to altered translation and synaptic plasticity in TSC mice.

## Methods

### Animals

Germline *Tsc2*^+/−^ mice were generated by crossing CMV-Cre (B6.C-Tg(CMV-cre)1Cgn/J) (JAX stock number: 006054) with Tsc2flox mice (JAX Stock number: 027458). F1 was then bred on the C57BL/6 J background to obtain WT and *Tsc2*^+/−^ mice. Animals were given ad libitum access to food and water and reared on a 12-h light–dark cycle. All experiments were approved by the Institutional Animal Care and Use Committee at University of Texas Southwestern and conducted in accordance with the National Institutes of Health Principles of Laboratory Animal Care.

### Hippocampal slice preparation

Acute hippocampal brain slices were prepared from P30-40 wild-type (WT) and *Tsc2*^+/−^ littermates as described previously [27]. Briefly, mice were anesthetized with ketamine (125 mg/kg)/xylazine (25 mg/kg) and transcardially perfused with chilled (4 °C) sucrose dissection buffer containing the following (in mm): 2.6 KCl, 1.25 NaH_2_PO_4_, 26 NaHCO_3_, 0.5 CaCl_2_, 5 MgCl_2_, 212 sucrose, and 10 dextrose aerated with 95% O_2_/5% CO_2_. Transverse hippocampal slices (400 µm) were obtained on a Leica VT1200S slicer. CA3 was cut off to avoid epileptogenic activity induced by DHPG. Slices were recovered for 3–4 h and maintained at 30 °C in artificial cerebral spinal fluid (ACSF) containing the following (in mM): 119 NaCl, 2.5 KCl, 2 CaCl_2_, 1 MgCl_2_, 26 NaHCO_3_, 1 NaH_2_PO_4_, and 11 D-glucose aerated with 95% O_2_/5% CO_2_ to pH 7.4. Slices were then treated with cycloheximide (100 µg/ml) in ACSF, 4 °C, snap-frozen on dry ice/EtOH bath, and stored at − 80 °C. Alternatively, hippocampal slices were treated with DHPG (100 µM) (Tocris) or artificial cerebrospinal fluid (ACSF, vehicle) for 5 min. For the cycloheximide experiments, hippocampal slices were pretreated with 75 nM cycloheximide for 45–60 min. At the end of the incubation, CA1 was dissected out, snap-frozen in a dry ice/ethanol bath and stored at − 80 °C. Some hippocampal slices were prepared without removing CA3 and used for RNA-seq and ribosome profiling experiments as indicated below (batches 1–5).

### Electrophysiology recordings

For all recordings, slices were submerged and perfused with ACSF at 2.5–3.5 ml/min (30 ± 1 °C). Field potentials (FPs) and EPSCs were evoked by stimulation of the Schaffer collateral pathway with a concentric bipolar tungsten electrode. Field potentials were recorded with a glass electrode (1 MΩ) filled with ACSF placed in the stratum radiatum of CA1. Test stimuli were delivered every 30 s, and a stable baseline was obtained at ∼50% of the maximum FP amplitude.

### Western Blotting

Western blotting was performed on whole hippocampus homogenates or on CA1 hippocampal slices, where indicated, and as previously described [14]. CA1 hippocampal slices were lysed with lysis buffer (50 mm Tris, pH 7.4, 120 mm NaCl, 50 mm NaF, and 1% Triton X-100, containing Protease Inhibitor Mixture, Sigma #P8340, and phosphatase inhibitor mixture 2 and 3, Sigma #P5726 and #P0040). Samples were homogenized using brief (5–10 s) pulses of sonication with an ultrasonic cell disruptor until lysates were clear. Lysates were then centrifuged at 15,700×*g* for 10 min at 4 °C, the pellets were discarded, and protein levels in the supernatant were measured using a Pierce BCA kit. After SDS–PAGE, proteins were transferred onto PVDF membranes. After blocking with 5% BSA in 1 × TBS, 0.05% Tween 20 for 1 h, membranes were incubated with the following primary antibodies in blocking buffer overnight at 4 °C: The following antibodies were used in this study: S6 Ribosomal Protein (Cell Signaling, #2217), Phospho-S6 Ribosomal Protein Ser235/236 (Cell Signaling, #4856), Akt (Cell Signaling, #9272), Phospho-Akt Ser473 (Cell Signaling, #4060), p44/42 MAPK (Erk1/2) (Cell Signaling #9102), Phospho-p44/42 MAPK (Erk1/2) Thr202/Tyr204 (Cell Signaling, #9101), eEF2 (Cell Signaling, #2332), Phospho-eEF2 Thr56 (Cell Signaling, #2331), mTOR (Cell Signaling, #2983), Phospho-mTOR Ser2448 (Cell Signaling, #2971), 4E-BP1 (Cell Signaling, #9452), Phospho-4E-BP1 Thr37/46 (Cell Signaling, #9459), RPL4 (Proteintech, #11302-1-AP), RPS25 (Sigma, #HPA031801-100UL), LARP1 (Proteintech, #13708-1-AP), FMRP (DSHB, 2F5-1), Glyceraldehyde-3-Phosphate Dehydrogenase (Millipore, #MAB374), actin (Millipore, #MAB1501). After three 10 min washes of 1 × TBS, 0.05% Tween 20, membranes were incubated with the appropriate HRP-conjugated secondary antibodies in 5% milk in 1 × TBS, 0.05% Tween 20 for 1 h at room temperature. After three 10 min washes of 1 × TBS, 0.05% Tween 20, the membranes were developed using ECL. For comparison of phosphorylated protein levels across conditions or genotypes, immunoreactive phosphorylated protein bands were normalized to total protein levels from the same slice homogenates (e.g., P-ERK/ERK), each of which was first normalized to loading control (either GAPDH or actin where indicated).

### Sample preparation for RiboSeq and RNA-Seq

Hippocampal slices from six mice were pooled, lysed in polysome buffer (as above but with the addition of 100 ug/mL cycloheximide, 25 U/mL Turbo Dnase I, 1 × EDTA-free protease inhibitor, 1% NP-40), and triturated with a 25G needle ten times. Following placement on ice for 10 min, the lysate was centrifuged 2000*g* for 10 min at 4 °C and the supernatant collected and centrifuged again for a 20,000*g* for 10 min at 4 °C. This supernatant was collected and the RNA concentration measured using the Qubit RNA HS Assay kit (Invitrogen, #Q32852). One-fourth of the lysate was saved for RNA-seq libraries (~ 1 ug of RNA), and the remainder (~ 3.5 ug) was treated with 11.3 ug of RNase A (Ambion, #AM2270) and 1410U of Rnase T1 (Thermo Fisher Scientific, #EN0542), which corresponds to a ratio of ~ 3ug Rnase A/375U Rnase T1 per ug of input RNA. The Rnase treatment was carried out at 25 °C for 30 min with gentle shaking and the digest stopped by placement on ice and the addition of 2.5u L of SuperRNaseIn (Invitrogen, #AM2696). The lysate was layered over a linear 10–50% sucrose gradient (prepared with polysome buffer supplemented with 1 mM DTT and 100 ug/mL CHX) and centrifuged at 35,000 rpm for 2.5 h at 4 °C in a SW41 rotor. The 80S monosome fractions were identified by continuous A260 monitoring and pooled for RNA extraction by TRIzol LS. To aid in RNA recovery of low concentration samples, 20 ug glycogen was added to all precipitation steps. A total of three biological replicates per genotype and treatment were made from hippocampal slices (CA1 enriched, batch 6–8) from *Tsc2*^+/−^ and their wild-type littermate mice. Wild-type slices had a total of eight biological replicates for RiboSeq and 7 for RNA-Seq (batch 1–5 from hippocampal slices with CA3, batch 6–8 from CA1 enriched).

### Library preparation

All RiboSeq libraries were prepared as previously described [28]. RiboZero Gold (Illumina, #MRZG12324) was used to deplete rRNA for RiboSeq libraries. The purified 80S monosome mRNA fragments were resolved on a 15% Tris-borate urea gel (National Diagnostics, #EC-833), and ribosome-protected fragments between 26 and 34 nt were recovered from the gel by the crush–soak method overnight in RNA extraction buffer (300 mM NaOAc pH5.5 1 mM EDTA 0.25% SDS) at 25 °C with constant horizontal rotation. The RNA was then precipitated, and the purified RNA was dephosphorylated with PNK enzyme (NEB, #M0201S) and ligated to preadenylated adaptors (IDT) using T4RNL2Tr.K227Q ligase (NEB, #M0351L). Reverse transcription (RT) was performed using SuperScript III (Invitrogen, #18080-044) in a modified 1X First Strand synthesis buffer without MgCl_2_ (50 mM Tris–HCl, pH 8.3, 75 mM KCl). Primers containing a 5 nt barcode and 8 nt unique molecular identifier (UMI) were used for RT. After RT, the RNA was hydrolyzed with a final concentration of 0.1 N NaOH for 20 min at 98 °C. The cDNA was resolved on a 10% TBU gel, and the 130–140 nt band was selected. The cDNA was eluted from crushed gel pieces overnight in DNA extraction buffer (300 mM NaCl, 1 mM EDTA, 10 mM Tris pH8.0) at 25 °C with constant horizontal rotation. The cDNA was circularized using CircLigase (Epicentre, #CL4115K) and depleted of tRNA and rRNA using biotinylated anti-sense probes (IDT) and Dynabeads MyOne Streptavidin C1 (Invitrogen, #65001). To determine the optimal number of PCR cycles, a test PCR was performed using the KAPA library amplification kit (Kapa Biosystems, #KK2611). The final PCR product was run on an 8% TBE gel, and the 180–190 nt bands were selected. The PCR product was eluted overnight in DNA extraction buffer as described earlier.

Batch 1–5 RNA-Seq libraries were prepared using RiboZero to deplete rRNA and then fragmented with 2X alkaline fragmentation solution (2 mM EDTA, 10 mM Na_2_CO_3_, 90 mM NaHCO_3_, pH ~ 9.3) at 95 °C for 15 min to yield ~ 140 nt fragments. The fragmented RNA was separated on a 10% TBU gel, and those migrating between 100 and 150 nt were selected. RT products were separated on a 10% TBU gel, and the 200–250 nt band was selected. The final PCR product was run on an 8% TBE gel, and the 260-300 bp band was selected. The size distribution of the final libraries was confirmed by Fragment Analyzer (UMMS Molecular Biology Core). Batch 6–8 RNA-Seq libraries were prepared using NEXTflex poly(A) beads (Bioo Scientific, #512980) and the NEXTFlex Rapid Directional qRNA-Seq kit (Bioo Scientific, # NOVA-5130-03D) according to manufacturer’s instructions.

The final libraries were purified using AMPure XP beads (Beckman Coulter, #A63880). Purified libraries were quantified by qPCR using KAPA Library Quantification kit (Kapa Biosystems, #KK4835) and were pooled to equimolar ratios. Libraries were sequenced on a NextSeq 500/550 High Output Kit v2 (Illumina, #FC-404-2005) as 75 bp single-end runs on a NextSeq500 sequencer.

### RiboSeq pipeline, read mapping, and differential expression analysis

**Fig. 1:**
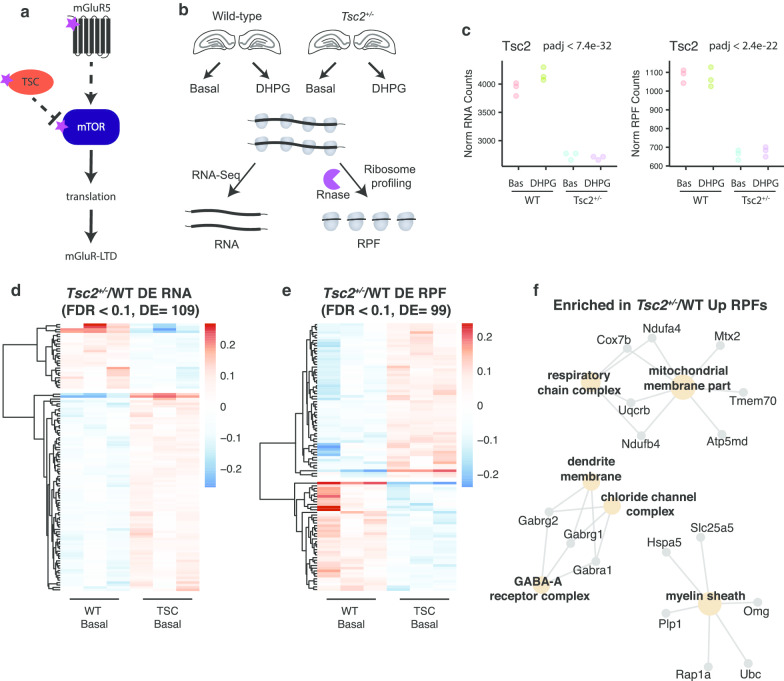
Ribosome profiling of *Tsc2*^+/−^ hippocampal slices reveals RNA and RPF dysregulation. **a** Salient features of TSC and mGluR5 signaling. mTOR indirectly receives negative and positive signals from TSC and mGluR5, respectively. mTOR promotes translation required for mGluR5-dependent long-term depression (LTD). Dashed and solid lines indicate indirect and direct effects, respectively. The purple stars indicate SFARI autism genes. **b** Schematic depicting the experimental design for RNA-Seq and ribosome profiling from CA1-enriched hippocampal slices of *Tsc2*^+/−^ and wild-type littermate controls. Slices were treated with DHPG (100 µM, 5 min) or artificial cerebrospinal fluid (vehicle) and then processed for RNA-Seq and ribosome profiling. Slices from six mice were pooled per genotype and condition, and three biological replicates were performed. RPF, ribosome-protected fragment. **c** Normalized count plots of RNA and RPF levels for *Tsc2* are shown (Wald-test for genotype comparison with BH correction. RNA: log2FC − 0.52, *p*adj < 7.4e−32; RPF log2FC − 0.70, *p*adj < 2.4e−22) **d**, **e** Heat maps of mRNAs (**d**) and RPFs (**e**) altered in *Tsc2*^+/−^ compared to WT under basal conditions. Relative gene expression is shown, and each column represents a biological replicate (*n* = 3) for RNA (DE = 109, FDR < 0.1) and RPFs (DE = 99, FDR < 0.1). **f** GO term enrichment of upregulated RPFs in *Tsc2*^+/−^ (*p*adj < 0.05)

The individual samples were separated based on 8 nt barcodes using custom scripts. Cutadapt (1.7.1) was used to remove the adapter (TGGAATTCTCGGGTGCCAAGGAGATCGGAAGAGCGGTTCAGCAGGAATGCCGAGACCG). The reads were then uploaded to the UMass Bioinformatics Core Dolphin platform for mapping (https://www.umassmed.edu/biocore/introducing-dolphin/). The reads were quality filtered using Trimmomatic (0.32) and then mapped to the rRNA and tRNA reference using bowtie2 (2.1.0). The rRNA and tRNA unmapped reads were kept and then mapped to mm10 using TopHat2 (2.0.9). Only uniquely mapped reads were kept, and PCR duplicates were marked based on UMI sequence and a custom script. SAMtools (0.019) was used to keep uniquely mapped reads without duplicates. For gene-level quantification of RiboSeq, RSEM/1.2.11 was used to align cleaned, unique reads to the RefSeq mm10 CDS with the first and last 30 nts removed to avoid quantifying initiation and termination peaks. For RNA-seq libraries, reads were aligned to the entire mm10 transcriptome. Counts from RSEM were batch-corrected using the Combat function in sva (3.24.4). Batch-corrected counts were used for DESeq2 (1.26.0) to identify differentially expressed genes for RNA and RiboSeq libraries. To validate our *Tsc2*^+/−^/WT differential expression results from Fig. 1, we performed 4990 random permutations of the sample labels without repeating the sample order. Differential expression was performed with these randomized sample labels using DESeq2 with identical parameters, design formula (genotype*treatment), and statistical cutoff (FDR < 0.1) as the real samples. The results of the randomizations are depicted in the histograms in Additional file 1: Fig S1. The majority of the analysis identified zero differentially expressed genes, and the randomizations yielded a mean of 14 DE RNAs and 11 DE RPFs, which is similar to the expected false positives from a FDR < 0.1 of our real data (11 false positives/109 DE RNAs and ten false positives/99 DE RPF). Normalized RNA and RPF counts from DESeq2 were used for generating plots. anota2seq (1.0.1) was used to calculate expression changes in TE (log2FC) using the relative log expression (RLE) for normalization.

### GO term, GSEA, and gene overlap analysis

GO term enrichment was performed using the R package clusterProfiler (3.14.0) [29]. For GSEA, mRNAs were ranked by their log2FoldChange and p value from DESeq2 output. Statistical significance was corrected with Benjamini-Hochberg, and terms with *p*adj < 0.05 were considered enriched. Gene overlap analysis was performed using the R package GeneOverlap (1.22.0). The genes past filtering from DE analysis were used as the background.

### Transcript feature analysis

Gene features were obtained from the UCSC Genome Browser using RefSeq annotations. For downstream transcript analysis, the most abundant isoform from the RSEM output of the RNA-Seq libraries was selected. ViennaRNA/RNAFold (2.1.6 h) was used to calculate the minimal free energy of the 5′UTR.

### Hippocampal TE calculation and rolling mean plots

The average CA1 translational efficiency (TE) was calculated by taking the ratio of RPF to RNA reads per kilobase per million mapped reads (RPKM) from three wild-type basal samples aligned to the mm10 CDS reference without the initiation and termination peak. For consistent TE calculations, mapping to the CDS for RPF and RNA was used, and transcripts longer than 300 nt and RPKM > 5 were included.

### Quantification and statistical analysis

**Fig. 2:**
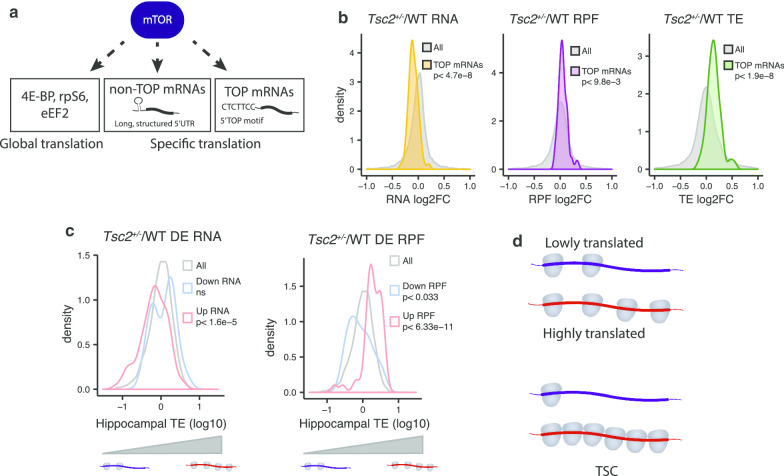
Dysregulation of TOP mRNAs in *Tsc2*^+/−^. **a** Schematic depicting mTOR control of translation. mTOR controls mRNA-specific translation via 5′ terminal oligopyrimidine (TOP) motifs or long, structured 5′UTRs. mTOR controls global initiation and elongation via phosphorylation/dephosphorylation of 4E-BP, rpS6, and eEF2. **b** mRNA, RPF, and translation efficiency (TE) expression changes (*Tsc2*^+/−^/WT log2FC) for TOP mRNAs (*n* = 68). Wilcoxon rank-sum test with correction by Bonferroni method. **c** Density plots of the hippocampal TE for the up and down RNA (left) and RPF (right) groups in *Tsc2*^+/−^/WT compared to all genes (one-tailed Kolmogorov–Smirnov test). The hippocampal TE is calculated from the average of wild-type basal slices as the ratio of RPF to RNA. **d** Schematic depiction of how loss of *Tsc2* could affect translation of lowly and highly translated mRNAs

**Fig. 3:**
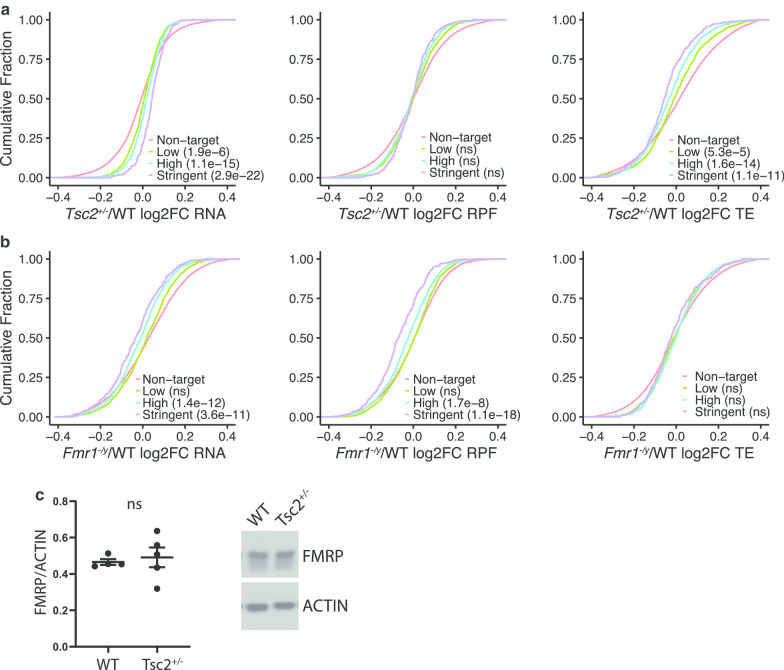
FMRP binding targets are regulated by TSC and FMRP in opposing directions. **a** Empirical cumulative distribution function (ECDF) of RNA (left), RPF (middle), and TE (right) expression changes in *Tsc2*^+/−^/WT (log2FC) of FMRP binding targets compared to nontarget mRNAs. FMRP binding targets are stratified into three groups based on binding stringency. Wilcoxon rank-sum test with correction by Bonferroni method. *p* values are indicated in the plot legend. **b** ECDF of RNA (left), RPF (middle), and TE (right) expression changes in *Fmr1*^*−/y*^/WT (log2FC) of FMRP targets compared to nontarget mRNAs. Data of hippocampal slices of *Fmr1*^*−/y*^ mice from Shah et al. [76]. Wilcoxon rank-sum test with correction by Bonferroni method. *p* values are indicated in the plot legend. **c** The levels of FMRP as determined by western blots of fresh, whole hippocampus from wild-type and *Tsc2*^+/−^ littermate. *n* = 5 animals, *ns* nonsignificant, unpaired *t* test

An unpaired Wilcoxon rank-sum test with Bonferroni correction was used to compare means between groups unless indicated otherwise in the text and figures. A one-tailed Kolmogorov–Smirnov test was used as shown in Fig. 2c. An unpaired t test was used for quantification of western blots in Fig. 3c and Additional file 1: Fig S2B, C. Western blot results in Fig. 6a–c were analyzed with a two-way, repeated-measures ANOVA. Factors are DHPG treatment and genotype, and repeated measures are basal and DHPG levels in the same mouse. *n* = # mice. Post hoc Sidak’s multiple comparison test was performed to determine statistical significance for specific comparisons, reported in the figure. For analysis of LTD results in Fig. 6f, g, we performed a linear mixed-effects model (LMM) statistic, whereby the variability due to random effects (animal, slice) was taken into account, allowing for direct measurement of genotype and/or treatment effects [30]. Mixed-effects models were fitted using IBM SPSS package. Fixed factors were genotype, treatment, genotype*treatment; repeated/random effects were slices nested within subject (mouse).

### Code availability

Custom scripts for generating plots will be available upon request to the lead contact.

## Results

### Translational profiling of the TSC hippocampus

The GTPase activating protein tuberous sclerosis complex (TSC) is an upstream but indirect repressor of mTOR, a kinase which forms the multi-subunit complex mTORC1 to stimulate translation in response to a number of signaling inputs including activated group I metabotropic glutamate receptors (e.g., mGluR5). mGluR5 activation leads to long-term depression (LTD), a protein synthesis-dependent form of synaptic plasticity frequently examined at hippocampal Schafer collateral-CA1 synapses (Fig. 1a) [12]. TSC is a neurodevelopmental disorder characterized by autism, epilepsy, and intellectual disability, and to study the role of TSC2 in synaptic plasticity, we generated mice lacking one copy of the *Tsc2* gene, which will henceforth be referred to as the *Tsc2*^+/−^ mouse.

To detect subtle changes in translation, we turned to ribosome profiling, a high-resolution and unbiased whole transcriptome method for determining the number and positions of ribosomes on mRNA. When combined with RNA-seq, the ratio of ribosome occupancy over input mRNA yields translational efficiency (TE) [23]. Although TE is often used as a proxy for protein synthesis, changes in TE may not always reflect protein output when ribosome elongation or mRNA levels are altered. Hippocampal slices, some of which were treated with dihydroxyphenylglycine (DHPG) for 5 min to induce LTD, were lysed in the presence of cycloheximide to prevent ribosome run-off, digested with RNase, and the 80S monomers containing ~ 30 nt ribosome-protected fragments (RPFs) were collected by ultracentrifugation (Fig. 1b). The isolated RPFs were appended with primers for library construction and sequencing (> 10 million uniquely mapped reads; libraries were strongly correlated with each other; Pearson’s *R* > 0.99).

*Tsc2* was the top differentially expressed mRNA at both the RNA and RPF levels, confirming the validity of our approach (Fig. 1c). Figure 1d, e shows heat maps of the RNAs and RPFs, respectively, from the hippocampal slices of the *Tsc2*^+/−^ mice and their littermate controls under basal (unstimulated) conditions that pass a significance cutoff of FDR < 0.1. The differentially expressed (DE) RNAs and RPFs altered in *Tsc2*^+/−^ had little overlap with each other (*n* = 5). The ratio of RPF to RNA yields translational efficiency (TE), but because changes in TE do not distinguish between RPF and RNA effects and because few RNAs passed statistical cutoff for changes in TE (log2FC), we considered RNA and RPF changes separately.

We observed enrichment for terms related to the GABA-receptor complex, mitochondrial membrane, and myelin sheath for RPFs that were Upregulated in the *Tsc2*^+/−^ brain (Fig. 1f, see Additional file 2: Table S1). No GO terms were enriched in the downregulated RPF group, likely due to the small number of genes. An altered excitatory/inhibitory balance is frequently observed in ASD, and the increased translation of GABA-A-related proteins may be a compensatory response to the increased hyperexcitability observed in *Tsc2*^+/−^ mice [31–35]. Altered translation of mitochondrial membrane proteins is consistent with the role of mTOR-dependent translational control of mitochondrial-related mRNAs and reports of mitochondrial dysfunction in *Tsc2*-deficient cultured neurons [36, 37]. The abnormalities in myelination in TSC patients and function of TSC/mTOR in myelination and function of oligodendrocytes [38, 39] may be due to translational control of myelin sheath proteins.

### Dysregulation of 5′ terminal oligopyrimidine (TOP) mRNAs

The major downstream substrate of TSC is mTOR, which controls specific and global translation (Fig. 2a). mRNAs that contain a 5′ terminal oligopyrimidine motif (TOP), which generally encode components of the translational machinery, and mRNAs with long, structured 5′ UTRs are especially sensitive to mTOR regulation [40–43]. TSC/mTOR also regulates general translation through phosphorylation/dephosphorylation events such as those that occur on 4E-BP, eIF4B, rpS6, and eEF2 [43–46]. Elevated mTOR signaling contributes to various behavioral and synaptic plasticity phenotypes in the brains of TSC mice as evidenced by their rescue by treatment with the mTOR inhibitor rapamycin [2, 24]. We found that the TOP mRNAs had mildly increased RPFs, but as a group, they had increased TE that is primarily driven by decreased RNA levels (Fig. 2b). mTOR can also promote initiation of non-TOP mRNAs that have long, structured 5′UTRs via eIF4E and eIF4A [40]. However, we find no difference in the 5′UTR lengths or predicted secondary structures in the up- and downregulated RPFs (Additional file 1: Fig S2A). To assess whether signaling events usually associated with mTOR activation occur in *Tsc2*^+/−^ hippocampus, we examined levels of phosphorylated mTOR, rpS6, and 4E-BP1. We also performed western blots for two TOP mRNAs, RPL4 AND RPS25, as well as LARP1, a TOP RNA-binding protein [47, 48]. Changes in the basal levels of these proteins are undetectable in the TSC hippocampus, and there is only a trend toward increases in phospho-4E-BP1 (Additional file 1: Fig S2B, C), similar to previous reports [35].

Because we did not identify specific mRNA features in the mRNAs and RPFs dysregulated in *Tsc2*^+/−^ mice, we wondered whether the mRNA-specific dysregulation may arise by altered global protein synthesis put forward by the “ribosome concentration” hypothesis from Mills and Green [49]. Their “ribosome concentration” hypothesis proposed that mRNAs with different rates of initiation respond bidirectionally to subtle changes in global translations from altered ribosome levels. Although we do not have evidence of altered mTORC1-rpS6 signaling or ribosome concentrations in *Tsc2*^+/−^ mice, we used this model as an intellectual framework for interpreting our results. We approximated initiation by calculating TE from wild-type basal slices, which will be referred to as hippocampal TE. The Up RNAs in *Tsc2*^+/−^ had a low hippocampal TE compared to all (Fig. 2c, left). We find that mRNAs with Down RPFs in *Tsc2*^+/−^ had a low hippocampal TE, and the Up RPFs had a high hippocampal TE compared to all (Fig. 2c, right). These results suggest that in *Tsc2*^+/−^ mice, poorly translated mRNAs are reduced even further, while highly translated mRNAs become more robustly translated (Fig. 2d). Finally, Additional file 1: Figure S1D shows transcript features that correlate with hippocampal TE. Consistent with another study, hippocampal TE is correlated with GC content of the CDS, 3′UTR, and 5′UTR and also CDS length [50].

### FMRP binding targets are altered in opposite directions in TSC and FXS mice

Loss of the RNA-binding protein Fragile X Mental Retardation Protein (FMRP) leads to Fragile X Syndrome (FXS). FMRP and TSC2 bidirectionally regulate mGluR-LTD and behavior, suggesting they coregulate similar mRNAs. In support of this, a combined TSC/FXS mouse rescues deficits in synaptic plasticity and learning to wild-type levels compared to the single mouse mutants [2]. Given the rescue paradigm of the FXS and TSC mouse, we determined whether FMRP targets are altered in *Tsc2*^+/−^ mice. We used a list of FMRP targets identified by CLIP (cross-link and immunoprecipitation) in CA1 pyramidal neurons, where FMRP targets were stratified into three groups based on binding strength: stringent (*n* = 327), high (*n* = 938), and low (*n* = 1330) binding targets [51]. FMRP targets are enriched (*p* < 5.3e−8) in the mRNAs altered in TSC (from Fig. 1d), and as a group, they have decreased TE that is driven by increased RNA levels that correlate with stringency of FMRP binding (Fig. 3a). FMRP targets have no change in RPFs in TSC (Fig. 3a, middle). Interestingly, the FMRP targets are decreased in the FXS mouse (*Fmr1*^*−/y*^), which is also correlated with stringency of FMRP binding (Fig. 3b) [51–54]. Because FMRP binding targets were dysregulated in *Tsc2*^+/−^, we determined whether FMRP protein levels were altered in *Tsc2*^+/−^. FMRP levels were unaltered in *Tsc2*^+/−^ (Fig. 3c), suggesting that the increased RNA levels of FMRP targets in *Tsc2*^+/−^ mice are not caused by altered FMRP levels. This bidirectional change in FMRP target mRNAs could be linked to the TSC/FXS mouse rescue paradigm and reveals a complex interaction between TSC2 and FMRP in steady-state regulation of mRNAs in the hippocampus.

### DHPG-induced mGluR signaling induces a rapid RNA and translational response

**Fig. 4:**
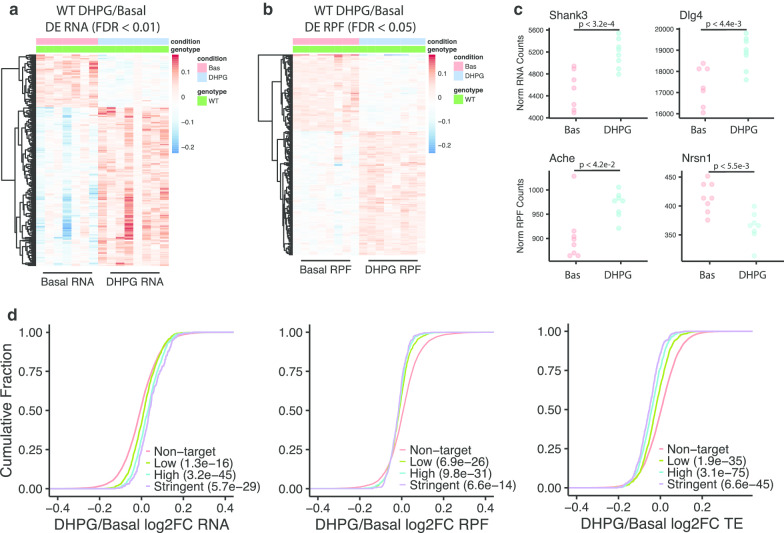
mGluR signaling induces rapid RNA and RPF changes in wild-type hippocampal slices. **a**, **b** Heat maps of mRNAs and RPFs that are responsive to DHPG (100 µM, 5 min) in wild-type hippocampal slices. Relative gene expression is shown; each column represents a biological replicate (*n* = 7 for RNA-Seq, *n* = 8 for ribosome profiling), differentially expressed RNA (*n* = 241, FDR < 0.01) and RPFs (*n* = 212, FDR < 0.05). **c** Examples of DHPG-responsive mRNAs (top) or RPFs (bottom) are shown with normalized RNA and RPF counts from DESeq2. *p* values are from the output of DESeq2 analysis (Wald-test with BH correction). **d** Empirical cumulative distribution function (ECDF) of RNA (left), RPF (middle), and TE (right) expression changes in DHPG/Basal (log2FC) of FMRP binding targets compared to nontarget mRNAs. FMRP binding targets are stratified into three groups based on binding stringency. Wilcoxon rank-sum test with correction by Bonferroni method. *p* values are indicated in the plot legend

mGluR-LTD is regulated by TSC, so to determine the identity of mGluR-stimulated translation of specific RNAs during LTD, we performed ribosome profiling and RNA-seq on hippocampal slices following 5 min of DHPG treatment [12, 18, 22]. Figure 4a, b shows that RNA (DE = 241, FDR < 0.01) and RPF (DE = 212, FDR < 0.05) levels undergo rapid changes in response to the mGluR1/5 agonist DHPG. DHPG-responsive RNAs and RPFs largely did not overlap (*n* = 5). Our DHPG-responsive translational changes in area CA1 do not overlap with those identified by polysome RNA-seq in cultured cortical neurons, which we attribute to different sample types and methodologies [21]. Gene set enrichment analysis reveals that GO terms related to G protein-coupled receptor activity, synaptic membranes, and the endoplasmic-reticulum membrane are enriched in the upregulated RNA and RPFs, and helicase activity is enriched in the downregulated RNAs and RPFs (Additional file 1: Fig S3A, B, Additional file 3: Table S2). Some GO terms are unique to the RNA or RPF changes. For example, the RNAs upregulated following DHPG are enriched for transcription-related functions, and the DHPG translational response is enriched for terms related to the lysosome. Some examples of DHPG-responsive mRNAs and RPFs that are high confidence SFARI autism genes (https://gene.sfari.org, score 1 or 2) are shown in Fig. 4c for *Shank3*, *Dlg4*, and *Ache*. *Dlg4* mRNA encodes PSD-95, which has increased mRNA and protein levels following DHPG-mGluR signaling [55]. *Nrsn1* mRNA encodes a neuron-enriched regulator of vesicle transport, which is translationally repressed 5 min after a hippocampus-dependent learning task [56]. Translational repression of *Nrsn1* mRNA during learning may be a requirement for this cognitive process as evidenced by the observation that its overexpression leads to deficits in object location and contextual fear memory. Because we did not observe immediate early genes (*c-fos*, *Arc*, and *Egr1*) in our list of differentially expressed RNAs, we infer that the rapid changes in RNA levels are likely due to alterations in RNA destruction and not transcription.

Because FMRP is regulated by and required for mGluR1/5 signaling to translation [57–60], we determined whether FMRP mRNA targets are responsive to this form of synaptic plasticity. FMRP binding targets are enriched in the DHPG-responsive DE RNAs (*p* < 1.8e−44) and RPFs (*p* < 5.0e−9). As a group, the FMRP targets have increased RNA levels following mGluR signaling that correlate with stringency of FMRP binding (Fig. 4d, left). FMRP targets have decreased RPFs following synaptic stimulation, and an overall decrease in TE that correlates with FMRP binding stringency (Fig. 4d, middle and right). Therefore, mGluR stimulation regulates translation and RNA levels of FMRP target mRNAs.

### mGluR-induced translational dysregulation in TSC

**Fig. 5:**
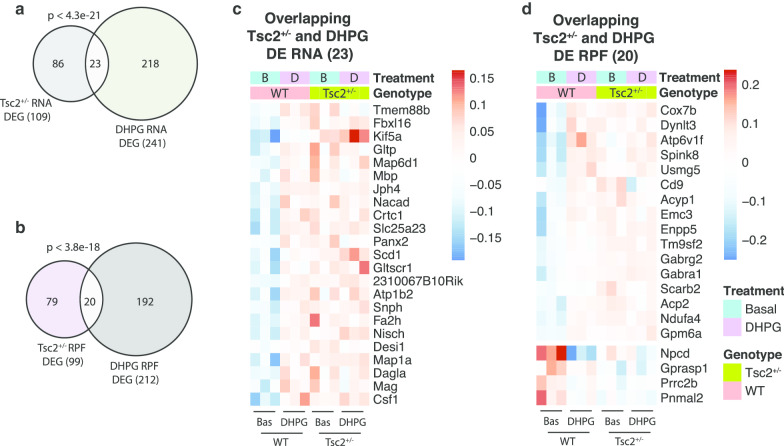
Some mRNAs altered in unstimulated *Tsc2*^+/−^ slices do not respond to DHPG-mGluR signaling. **a**, **b** Venn diagrams showing overlap between *Tsc2*^+/−^ dysregulated RNAs and DHPG-responsive RNAs (**a**) and *Tsc2*^+/−^ dysregulated RPFs and DHPG-responsive RPFs (**b**). Gene overlap test was performed using Fisher’s exact test and a background size of expressed RNAs used in DE analysis. **c**, **d** Heat maps of mRNAs (**c**) and RPFs (**d**) that overlap from (**a**) and (**b**). Relative gene expression is shown, and each column represents a biological replicate of littermates (*n* = 3)

*Tsc2*^+/−^ mice have deficient mGluR-LTD, so we determined whether the RNA and translational response after DHPG is altered. We lacked sufficient statistical power to detect genotype-dependent alterations in the DHPG response in *Tsc2*^+/−^ slices with their littermate controls. Instead, we compared the genes altered in *Tsc2*^+/−^ (Fig. 1d, e) and the DHPG-responsive genes in wild type (Fig. 4a, b) and found a statistically significant overlap for the RNAs (*n* = 23, *p* < 4.3e−21) and RPFs (*n* = 20, *p* < 3.8e−18) (Fig. 5a, b; Additional file 4). Interestingly, 16 of the 23 RNAs that overlap between *Tsc2*^+/−^ and mGluR1/5-stimulated are FMRP mRNA targets (e.g., *Kif5a*, *Snph*, *Nisch*, and *Slc25a23*). No GO terms were enriched in the overlapping list, likely due to the small number of genes. Heat maps of the overlapped mRNAs and RPFs in Fig. 4a, b show that most of the upregulated mRNAs and RPFs in unstimulated *Tsc2*^+/−^ slices appear to have a deficient DHPG response compared to wild-type slices (Fig. 5c, d). Our results suggest that some mRNAs and RPFs that are elevated in unstimulated *Tsc2*^+/−^ slices cannot respond to DHPG.

### Signaling to eukaryotic elongation factor 2 is altered in the *Tsc2*^+/−^ hippocampus

**Fig. 6:**
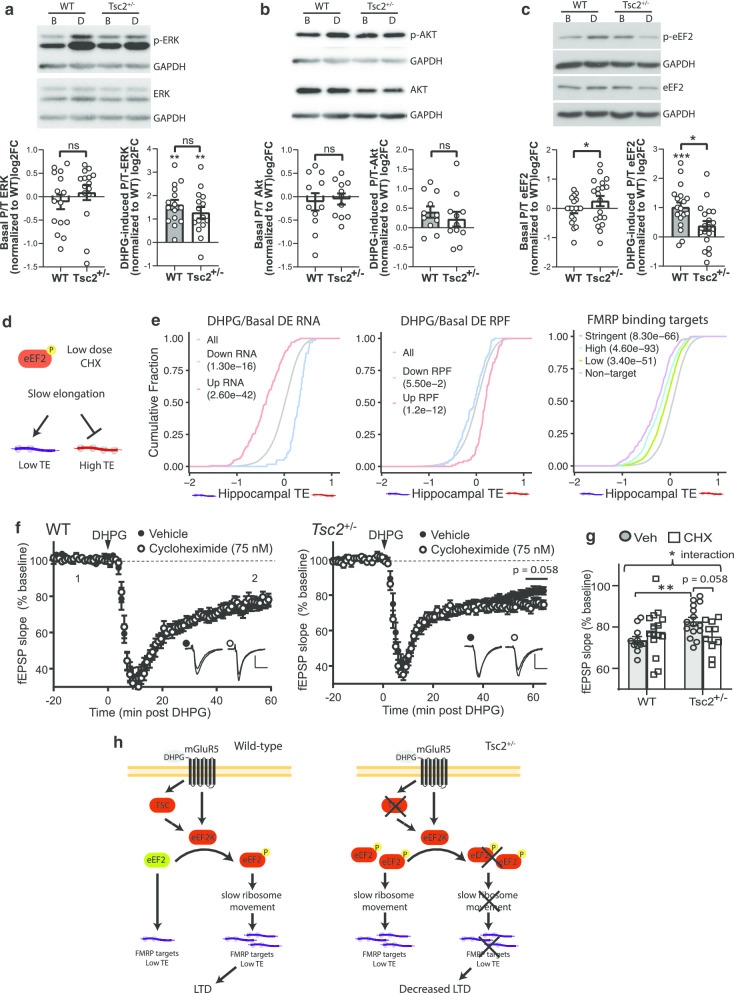
Deficient mGluR1/5-regulated signaling to translation elongation suppresses mGluR-LTD in *Tsc2*^+/−^ mice. **a**–**c** The levels of phosphorylated (P) and total (T) ERK (**a**), AKT (**b**), and eEF2 (**c**) in CA1-enriched hippocampal slices from wild-type and *Tsc2*^+/−^ mouse littermates as determined by western blots under basal or DHPG-treatment condition (100 µM; 5 min). *p < 0.05; **p < 0.01; ***p < 0.001; 2 way ANOVA, post hoc Sidak’s test, *n* = 11–19 mice/genotype, 4–5 slices per drug condition/mouse. Asterisks below brackets indicate an effect of DHPG on phosphoprotein levels within genotype. Asterisks above brackets indicate effects between genotypes. **d** Schematic depicting that global slowing of ribosome movement through eEF2 phosphorylation or low dose cycloheximide (CHX) may promote protein synthesis of lowly translated mRNAs while inhibiting highly translated mRNAs. **e** Empirical cumulative distribution function (ECDF) plot of hippocampal TE for the Up and Down RNAs (left) and RPFs (middle) in DHPG/basal compared to all genes. Right: ECDF plot of hippocampal TE for FMRP binding targets compared to non-target mRNAs. FMRP binding targets are stratified into three groups based on binding stringency. Wilcoxon rank-sum test with correction by Bonferroni method. *p* values are indicated in the plot legend. **f** Left: Time course demonstrating that brief DHPG application (100 µM; 5 min) induces long-term synaptic depression (LTD) of field (f) EPSP slopes in WT hippocampal slices that is unaffected by low doses of cycloheximide (45–60 min pretreatment, 75 nM; *n* = 12 and 15 slices; 6–7 mice/condition). Right: In *Tsc2*^+/−^ mice pretreatment with low-dose cycloheximide enhances LTD magnitude (*n* = 10 and 15 slices; 7 mice/condition). Plotted are group averages of fEPSP slope (mean ± SEM) normalized to pre-DHPG baseline as a function of time. Inset: Example fEPSP from baseline and during LTD (55–60 min post DHPG). Scale = 0.5 mV/10 ms. **g** LTD magnitude, measured at 55–60 min post-DHPG application in vehicle and cycloheximide in each genotype. There is a significant interaction between cycloheximide and *Tsc2* genotype (*F*(1,47) = 5.197, *p* < 0.05; LMM). With post hoc pairwise comparisons, there is a strong trend for cycloheximide to enhance LTD in *Tsc2*^+/−^ mice (veh: 83 ± 2% of baseline; cycloheximide; 75 ± 5%; *n* = 15 and ten slices from four mice/treatment; *F*(1,23) = 5.445, *p* = 0.058; LMM) but not WT mice (veh: 74 ± 2% of baseline; cycloheximide; 77 ± 3%; *n* = 11 and 15 slices from 6 and 5 mice, respectively/treatment; *F*(1,24) = 3.819, *p* = 0.14; LMM). In vehicle, LTD is reduced in *Tsc2*^+/−^ mice in comparison with WT, but not in low-dose cycloheximide. (*p < 0.05; **p < 0.01). **h** Schematic showing DHPG induction of eEF2 phosphorylation in hippocampal slices. In WT slices, DHPG leads to increased levels of phospho-eEF2 through signaling to eEF2K, which presumably slows ribosome transit and promotes expression of lowly translated mRNAs required for mGluR-LTD, such as FMRP binding targets. In TSC slices, levels of phospho-eEF2 are elevated basally and correlate with increased mRNA abundance of FMRP binding targets. DHPG treatment does not further increase eEF2 phosphorylation, and *Tsc2*^+/−^ slices may have excessive ribosome transit during mGluR signaling that contributes to deficient mGluR-LTD.

The sequencing results of the *Tsc2*^+/−^ slices under basal and DHPG conditions suggest there is a deficiency in the ability of DHPG to regulate translation in the *Tsc2*^+/−^ mice. To determine whether mGluR1/5 signaling to translational control was altered in *Tsc2*^+/−^, hippocampal slices were treated with DHPG followed by western blotting for several well-established downstream substrates of mGluR activation. Figure 6a, b shows that under basal conditions, phosphorylated (phospho) ERK and AKT were unaffected by loss of one allele of Tsc2. In response to DHPG treatment, phospho-ERK levels were similarly elevated in WT and *Tsc2*^+/−^ slices, consistent with other observations [2, 42]. DHPG did not induce phospho-Akt in either WT or *Tsc2*^+/−^ slices. Because both mTOR signaling and mGluR5 signaling regulate phosphorylation of eukaryotic elongation factor 2 (eEF2), we determined whether signaling to eEF2 could be altered. In wild-type slices, DHPG treatment robustly induces eEF2 phosphorylation (Fig. 6c). Surprisingly, *Tsc2*^+/−^ slices have elevated phospho-eEF2 basally, and DHPG treatment fails to induce eEF2 phosphorylation. Total levels of eEF2 are unchanged in *Tsc2*^+/−^ at basal (106 ± 6% of WT; *n* = 19 mice; n.s.; 2way ANOVA, post hoc Sidak’s test) and following DHPG treatment (WT 101.7 ± 8.02%, *Tsc2*^+/−^ 114.4 ± 9.69%, *n* = 19, n.s.; 2way ANOVA, post hoc Sidak’s test).

eEF2 is a GTP-dependent elongation factor that promotes transfer of peptidyl-tRNAs from the A to P site of the ribosome. Phosphorylation of eEF2 on threonine 56 prevents it from binding to ribosomes, leading to inhibition of translation elongation [61]. Somewhat counterintuitively, eEF2 kinase and its phosphorylation of eEF2 are necessary for mGluR-induced synthesis of proteins required for LTD [18]. Global inhibition of translation elongation, such as through phospho-eEF2 or cycloheximide (a chemical inhibitor of ribosome translocation), may promote synthesis of poorly translated mRNAs by liberating rate-limiting translation factors (Fig. 6d) [18, 62, 63]. Thus, we determined whether the mRNAs responsive to mGluR-LTD signaling are enriched for poorly translated mRNAs by using hippocampal translation efficiency as described earlier. We find that the Up RNA group after DHPG treatment has low basal translation efficiency compared to unchanged mRNAs and the Down mRNA group has high basal translation efficiency (Fig. 6e, left). For the translational response, the Up RPF group after DHPG-mGluR signaling has high basal translation efficiency compared to other mRNAs, whereas the Down RPF group had the same translation efficiency as the unchanged mRNAs (Fig. 6e, middle). Because DHPG-mGluR signaling may reduce ribosome translocation via phospho-eEF2, it is possible that at least some of the mRNAs with increased RPFs are associated with stalled ribosomes, in which case they would have decreased translation. Because FMRP binding targets are enriched in the DHPG-mGluR-responsive Up mRNA group, we predicted that FMRP targets are also lowly translated in the hippocampus. Indeed, FMRP binding targets have low basal translation efficiency compared to nontargets (Fig. 6e, right). Interestingly, the strength of FMRP binding negatively correlates with basal translation efficiency where the most stringent FMRP targets have lower basal translation efficiency compared to the low- and high-stringency groups. Taken together, these observations suggest that mGluR5 signaling increases expression of mRNAs that are lowly translated, such as FMRP binding targets.

Because *Tsc2*^+/−^ slices have a deficient phospho-eEF2 response to DHPG treatment, we hypothesized that ribosome elongation is excessive during mGluR signaling in *Tsc2*^+/−^ and may contribute to deficient mGluR-LTD. We used a low concentrations of cycloheximide (75 nM), which inhibits ribosome elongation by binding to the E-site of the ribosome to prevent eEF2-mediated translocation [64]. This concentration of cycloheximide restored mGluR-LTD in eEF2K knockout mice without affecting mGluR-LTD in WT mice [18]. We induced mGluR-LTD with DHPG in hippocampal slices and tested the effects of low-dose cycloheximide (75 nM) to inhibit ribosome elongation. While there were no main effects of genotype or treatment overall on LTD magnitude, we observed a significant interaction of genotype*treatment (Fig. 6f, g). With post hoc pairwise comparisons, there was a strong trend for cycloheximide to enhance mGluR-LTD in *Tsc2*^+/−^ (veh: 83 ± 2% of baseline; cycloheximide; 75 ± 5%) but not WT mice (veh: 74 ± 2% of baseline; cycloheximide; 77 ± 3%). In vehicle, mGluR-LTD in *Tsc2*^+/−^ was less than WT as previously reported [2]. Our data suggest that the deficiency of mGluR1/5 induced P-eEF2 and suppression of translation elongation in *Tsc2*^+*/*^ mice limits LTD magnitude and this can be reinstated by pharmacological inhibition of ribosome elongation (Fig. 6h).

## Discussion

Using ribosome profiling and RNA-seq, we identified changes in ribosome footprint number and RNA levels in *Tsc2*^+/−^ hippocampal slices and following mGluR-LTD. Our experimental design captures changes from multiple cell types, which is reflective of the tissue complexity of the hippocampus. We found translation of myelin-related mRNAs increased in *Tsc2*^+/−^ mice, which are predominantly found in oligodendrocytes. The tissue complexity may explain why we did not capture translation of known DHPG-induced mRNAs Arc and Map1b, which are translated following DHPG treatment in dendrites of excitatory neurons [22, 65].

In complete loss of TSC1 or TSC2 models, there is clearly excessive mTOR activity that leads to widespread changes in translation [66]. However, mTOR hyperactivation is not always as obvious in *Tsc1* and *Tsc2* heterozygote models in the brain [35]. Using a *Tsc2*^+/−^ model, we do not observe signs of mTOR hyperactivation by western blot of phosphorylated mTOR, 4E-BP1, and rpS6 in the hippocampus. Instead, we find evidence of disrupted global mRNA translation by increased TE of TOP mRNAs and elevated levels of phosphorylated eEF2. The TOP mRNAs, the most well-studied TSC/mTOR translational targets, have increased TE from predominantly decreased RNA and increased RPF levels. The decreased RNA levels of the TOP mRNAs are surprising, given that one predicts increased expression of TOP mRNAs from mTOR hyperactivity from loss of TSC2. There could be two possibilities to explain this observation. This may be an example of translational buffering to maintain homeostasis of proteins encoded by TOP mRNAs, where RNA levels decrease to offset the increase in ribosome occupancy [67, 68]. Alternatively, the decreased RNA levels of TOP mRNAs may be reflective of the altered signaling to phospho-eEF2, which is increased and opposite of the direction that is predicted by activation of mTORC1-S6K-eEF2K signaling in *Tsc2*^+/−^ [45, 46]. The increased in basal phosphorylated eEF2 in *Tsc2*^+/−^mice may occur via other effectors to eEF2K, such as increased intracellular Ca2 + and mGluR1/5 signaling [18, 69]. Our finding of increased basal levels of phospho-eEF2 is consistent with the observation of decreased protein synthesis rates in hippocampal slices of *Tsc2*^+/−^ mice [2]. Our study suggests a more complex role of translational dysregulation in the *Tsc2*^+/−^ hippocampus outside of simple mTORC1-rpS6 hyperactivation. Given the many downstream substrates of mTOR, it is possible that some of the mRNA and translational alterations observed in *Tsc2*^+/−^ are through mTORC1-dependent processes outside of translation (e.g., autophagy, lipogenesis) or mTORC2. The small sample size for the *Tsc2* genotype comparison is a limitation to our sequencing analysis, which has low power to identify differences from lowly expressed genes and small effect changes frequently observed in the brain and neuropsychiatric disorders. Our results do not rule out more dramatic, cell-type specific arising from *Tsc2* haploinsufficiency, such as in excitatory neurons. These results also highlight the importance of maintaining appropriate gene dosage in modeling TSC and other mTORopathies such as PTEN, where only one allele is usually mutated in patients.

Because *Tsc2*^+/−^ mice have decreased protein synthesis in the hippocampus and DHPG-mGluR1/5 signaling can activate near global regulators of translation, and we used the “ribosome concentration” hypothesis as an intellectual framework for interpreting our results. This model was originally proposed to explain the seemingly mRNA-specific effects arising from ribosomopathies, a group of disorders with mutations in ribosomal proteins that manifest with cell-type-specific features. Using mathematical modeling and assuming initiation as the rate-limiting step of translation, the “ribosome concentration” hypothesis predicts that efficiently and inefficiently translated mRNAs respond bidirectionally to global decreases in translation arising from reduced levels of ribosomes, and recent experimental evidence from yeast and hematopoietic cells supports this hypothesis, albeit in a direction opposite of originally predicted [70, 71]. Here, we observe a trend where in *Tsc2*^+/−^ mice, inefficiently translated mRNAs have decreased translation and efficiently translated mRNAs have increased translation (Fig. 2e). Surprisingly, during DHPG-mGluR1/5 signaling, the degree of translation efficiency correlates more with RNA-level changes rather than translation, and FMRP binding targets have low translation efficiency compared to all genes (Fig. 6e, left). The factors that regulate this phenomenon in *Tsc2*^+/−^ mice and DHPG-mGluR1/5 signaling remain unknown.

RNA-seq reveals that mGluR activation induces a rapid response in both increased and decreased mRNA levels. Because translation is often linked to RNA stabilization/destabilization, this mGluR response could be linked to this process [72]. The decrease in specific mRNAs may be the result of heightened rates of degradation, which could be the consequence of mGluR-dependent upregulation of microRNAs [73]. For mRNAs that increase, there may be a constant rate of mRNA turnover in hippocampal tissue, and mGluR activation could lead to mRNA stabilization through regulation of translation or microRNA dissociation [60]. The DHPG-mGluR-responsive mRNAs are highly enriched for FMRP targets and genes encoding synaptic proteins. For example, our study and that of Zalfa et al. [55] found that levels of PSD-95 mRNA (*Dlg4*), an FMRP target, increase after mGluR activation in cultured neurons and are regulated by interaction with a microRNA [60]. Several recent studies have supported the role of FMRP in modulating stability of its targets through N^6^-methyladenosine modification of the mRNA and codon optimality [54, 74]. The observation that DHPG results in decreased RPFs and TE on FMRP target mRNAs suggests they have reduced translation. This would be a surprising finding based on previous work that DHPG rapidly stimulates the synthesis of proteins encoded by FMRP target mRNAs such as Map1b, Arc, EF1a, and PSD-95 [22, 60, 65, 75]. Therefore, decreased RPF and TE may not always indicate reduced synthesis of protein. Some evidence indicates that FMRP stalls or slows ribosome translocation, and mGluR stimulation regulates translation elongation through FMRP and eEF2 phosphorylation [18, 59, 76, 77]. Thus, for some mRNAs, the observed decrease in RPF or TE on FMRP target mRNAs in response to DHPG may be a consequence of enhanced ribosome movement that reflects increased protein synthesis.

Recent work has revealed regulation of translational elongation as critical for multiple forms of protein-synthesis-dependent synaptic plasticity, including mGluR-LTD signaling [18, 78–80]. DHPG-induced mGluR1/5 signaling via ERK and AKT is normal in the *Tsc2*^+/−^ hippocampus, which mainly feeds into regulation of translation initiation [81]. Instead, signaling to ribosome translocation (polypeptide elongation) is altered in *Tsc2*^+/−^ and a low-dose cycloheximide enhances mGluR-LTD in these mice. Increased levels of phospho-eEF2 and low-dose cycloheximide have previously been shown to paradoxically increase translation of specific mRNAs, such as Arc and alpha-CAMKII, despite globally inhibiting protein synthesis [18, 80]. Furthermore, we find evidence for a mechanism of occlusion via eEF2 phosphorylation in *Tsc2*^+/−^, where some of the mRNAs and RPFs increased in *Tsc2*^+/−^ mice appear to no longer respond to DHPG, consistent with the higher basal levels of eEF2 and the deficient eEF2 response following DHPG. Our work suggests a novel mechanism of translational dysregulation in *Tsc2*^+/−^ and mGluR synaptic plasticity through signaling to ribosome elongation.

A combined *Tsc2*^+/−^ and *Fmr1*^*−/y*^ mouse rescues phenotypes in synaptic plasticity and contextual fear conditioning in each other, yet the mechanism of this rescue paradigm has remained elusive. In *Tsc2*^+/−^ and following DHPG-mGluR signaling, FMRP binding targets have decreased TE from increased RNA levels; in *Fmr1*^*−/y*^, RNA levels of FMRP targets are instead decreased. Furthermore, DHPG-induced phospho-eEF2 is deficient in *Tsc2*^+/−^, but enhanced in *Fmr1*^*−/y*^ mice [14]. This bidirectional alteration of FMRP binding targets and mGluR5 signaling to elongation suggest mechanisms for the opposing changes in synaptic plasticity and rescue paradigm of the *Tsc2*^+/−^/*Fmr1*^*−/y*^ mice. Because total FMRP protein levels are unchanged in *Tsc2*^+/−^, the dysregulation of FMRP binding targets may be through alternate pathways in transcription, mRNA stability, and/or translation. Furthermore, mRNAs that are dysregulated in *Tsc2*^+/−^ are enriched for DHPG-mGluR-responsive mRNAs and many of these overlapping mRNAs are FMRP binding targets. These results reveal a complex interaction between TSC2 and FMRP in steady state and mGluR regulation of distinct mRNA subclasses.

### Limitations

We anticipated that loss of one copy of *Tsc2* would result in widespread but perhaps subtle gene expression changes, consistent with other mouse models of neuropsychiatric disorders. As such, we used a FDR *p*-adjusted cutoff of 0.1 for the genotype comparison. One limitation of the interpretation of our ribosome profiling results is that we identified altered signaling to ribosome movement in *Tsc2*^+/−^ and following mGluR-LTD signaling. Thus, changes in ribosome density may not a priori reflect changes in protein synthesis. Multiple mouse models of autism have altered mGluR-LTD signaling, but evidence of altered mGluR-LTD in humans with autism has yet to be established. For Figs. 1 and 5, we utilized hippocampal slices prepared from 72 mice for a sample size of three per group, a standard practice in the ribosome profiling and RNA-seq field. Sequencing experiments with a sample size of 3 are limited by low power, and to reach a power of > 0.8 would require a sample size of 30 libraries from over 600 littermate mice, which is prohibitive in cost for animals and reagents. The low power indicates that our datasets have a high probability of Type II errors (false negatives), and thus we would be unlikely to detect a genotype effect. This caveat, however, does not change the conclusions of our study. In any event, our findings need to be replicated in other studies.

## Conclusions

By performing RNA-seq and ribosome profiling on a *Tsc2*^+/−^ mouse model and following induction of mGluR-LTD, we identified changes in FMRP binding targets that suggest a molecular basis for bidirectional regulation of synaptic plasticity and behavior by TSC2 and FMRP. Our study also suggests that altered mGluR-regulated translation elongation contributes to impaired synaptic plasticity in *Tsc2*^+/−^ mice.

## Supplementary information

**Additional file 1: Fig. S1**. Validation of differential expression results from *Tsc2*^+/−^ versus wild type from Fig. 1. Histogram of the number of differentially expressed genes called from 4990 random permutations of sample labels for the genotype comparison from **a** RNA-seq and **b** ribosome profiling. **Fig. S2**. **a** Density plot of the length (left) and minimal free energy (MFE) (right) of the 5′UTR of Down and Up RPFs in *Tsc2*^+/−^ compared to all mRNAs. *ns* nonsignificant, Wilcoxon rank-sum test with correction by Bonferroni method. **b** The ratios of phosphorylated/total mTOR, rpS6, and 4EBP1 as determined by western blots of whole hippocampal lysates are unchanged in *Tsc2*^+/−^ mice as compared to wild-type littermates (*n* = 13–15 mice/genotype, *ns* non-significant, unpaired *t* test). **c** Total levels of RPL4, RPS25, and LARP1 from hippocampus of wild-type and *Tsc2*^+/−^ littermates (*n* = 13–15 mice/genotype, ns = nonsignificant, unpaired *t* test). **d** Pearson correlation coefficient was computed for pairs of transcript features including translation efficiency (TE), coding length, and GC content (CDS), 5′ untranslated region length and GC content (5′UTR), 3′ untranslated region length and GC content (3′UTR), and RNA abundance (TPM, transcripts per million). Red indicates a positive correlation, and blue a negative correlation. Gray indicates a correlation of identical parameters. *p* < 0.05 for all correlation coefficients. **Fig. S3**. Gene set enrichment analysis of mGluR-responsive RNAs and translation. **a**, **b** Gene set enrichment analysis of GO terms enriched (*p*adj < 0.05) in DHPG/Basal RNAs (A) and RPFs (B). Gene lists were ranked by wild-type DHPG/Basal expression changes (log2FC) and *p* value. GO terms are separated by enrichment in either upregulated (increased) or downregulated (decreased) RNAs and RPFs.

**Additional file 2: Table S1**. Table of GO terms enriched in *Tsc2*^+/−^/WT upregulated RNA and RPFs from Fig. 1D, E (*p*adj < 0.05 for cellular compartment, molecular function, and biological process).

**Additional file 3: Table S2**. Table of GO terms enriched in wild-type DHPG/basal ranked list of RNA and RPFs expression changes from Supplemental Fig. 2 (*p*adj < 0.05 for cellular compartment, molecular function, and biological process).

**Additional file 4: Table S3**. Table of DESeq2 output for RNA and RPF expression changes for *Tsc2*^+/−^/WT and WT DHPG/Basal comparisons. FMRP binding targets are indicated in the last column based on binding strength (stringent, high, low). Nontargets are indicated by nonclip. Overlapping mRNAs and RPF altered in *Tsc2*^+/−^ and responsive to DHPG from Fig. 5 are listed.

**Additional file 5: Table S4**. Table of anota2seq output for RNA, RPF, and TE expression changes for *Tsc2*^+/−^/WT. The delta tab has calculated expression changes (log2FC) for RNA, RPF, and TE. The total mRNA tab has analysis for RNA expression changes (log2FC). The translated mRNA tab has analysis for RPF expression changes (log2FC). The buffering tab has analysis for TE expression changes (log2FC) that likely leads to translational buffering. The translation tab has analysis for TE expression changes (log2FC) that likely leads to altered protein levels.

**Additional file 6: Table S5**. Table of anota2seq output for RNA, RPF, and TE expression changes for WT DHPG/Basal. The delta tab has calculated expression changes (log2FC) for RNA, RPF, and TE. The total mRNA tab has analysis for RNA expression changes (log2FC). The translated mRNA tab has analysis for RPF expression changes (log2FC). The buffering tab has analysis for TE expression changes (log2FC) that likely leads to translational buffering. The translation tab has analysis for TE expression changes (log2FC) that likely leads to altered protein levels.

## Data Availability

The datasets supporting the conclusions of this article are included within the article in Additional files 4, 5, 6. The RNA-seq and Ribo-seq datasets supporting the conclusions of this article are available in the GEO Database repository: GSE144539 and GSE158881. Publically available datasets analyzed in the current study are from the following GEO Database repository: Shah et al., 2020: GSE143333.

## Abbreviations

CHX: Cycloheximide
DE: Differentially expressed
DHPG: Dihydroxyphenylglycine
eEF2: Eukaryotic elongation factor 2
FMRP: Fragile X Mental Retardation Protein
LTD: Long-term depression
mGluR: Metabotropic glutamate receptor
mTOR: Mechanistic target of rapamycin
RPF: Ribosome-protected fragment
TE: Translation efficiency
TOP: Terminal oligopyrimidine tract
TSC: Tuberous sclerosis complex
WT: Wild type
