# Supplementary figures and images for: Ribosome profiling in mouse hippocampus: plasticity-induced regulation and bidirectional control by TSC2 and FMRP
Source: Mol Autism. 2020 Oct 14;11:78. doi: 10.1186/s13229-020-00384-9 (PMC7556950; doi:10.1186/s13229-020-00384-9)

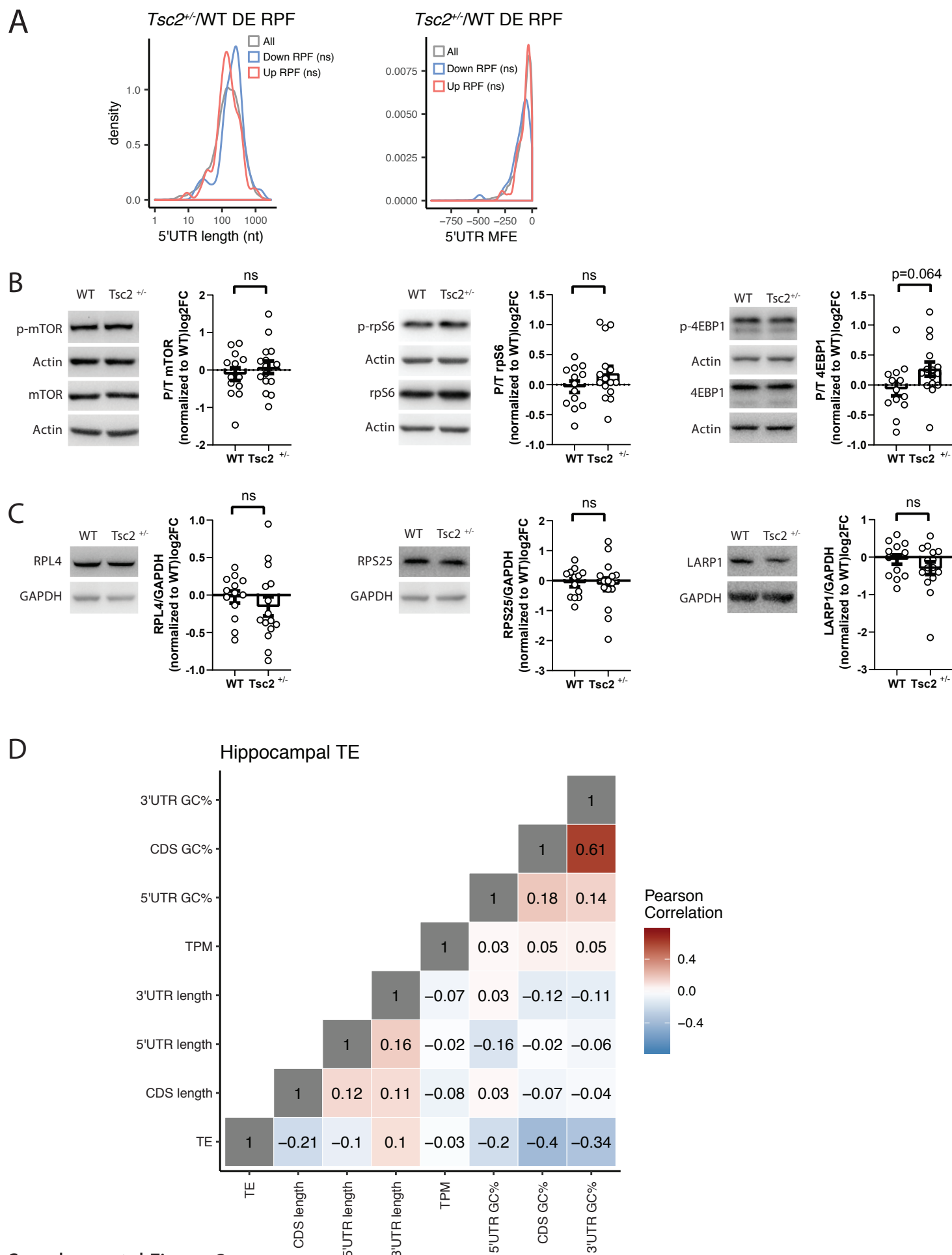

Supplemental Figure 2

A

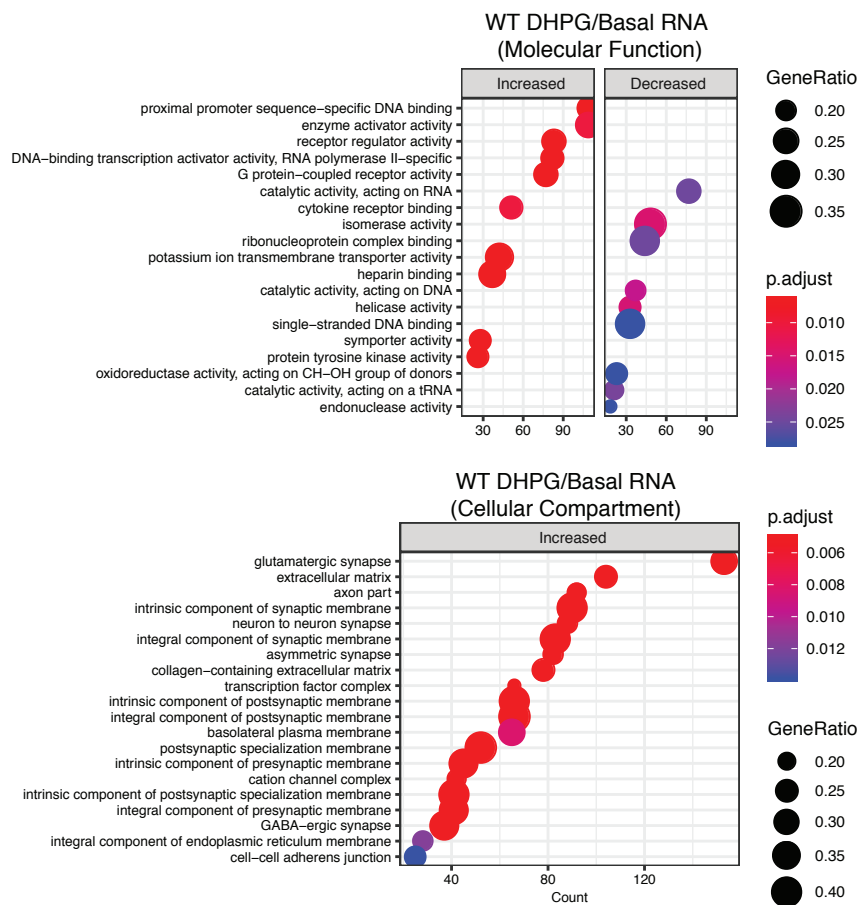

B

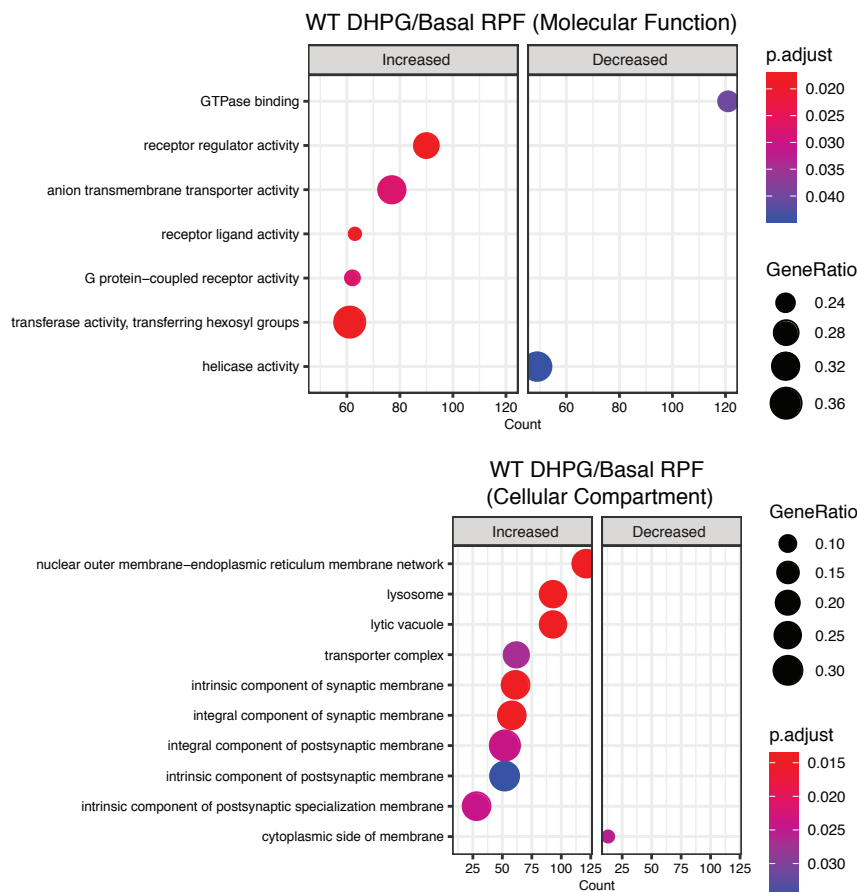

Supplemental Figure 3

Supplement: Supplementary file 1 — Additional file 1: Fig. S1. Validation of differential expression results from Tsc2+/− versus wild type from Fig. 1. Histogram of the number of differentially expressed genes called from 4990 random permutations of sample labels for the genotype comparison from a RNA-seq and b ribosome profiling. Fig. S2. a Density plot of the length (left) and minimal free energy (MFE) (right) of the 5′UTR of Down and Up RPFs in Tsc2+/− compared to all mRNAs. ns nonsignificant, Wilcoxon rank-sum test with correction by Bonferroni method. b The ratios of phosphorylated/total mTOR, rpS6, and 4EBP1 as determined by western blots of whole hippocampal lysates are unchanged in Tsc2+/− mice as compared to wild-type littermates (n = 13–15 mice/genotype, ns non-significant, unpaired t test). c Total levels of RPL4, RPS25, and LARP1 from hippocampus of wild-type and Tsc2+/− littermates (n = 13–15 mice/genotype, ns = nonsignificant, unpaired t test). d Pearson correlation coefficient was computed for pairs of transcript features including translation efficiency (TE), coding length, and GC content (CDS), 5′ untranslated region length and GC content (5′UTR), 3′ untranslated region length and GC content (3′UTR), and RNA abundance (TPM, transcripts per million). Red indicates a positive correlation, and blue a negative correlation. Gray indicates a correlation of identical parameters. p < 0.05 for all correlation coefficients. Fig. S3. Gene set enrichment analysis of mGluR-responsive RNAs and translation. a, b Gene set enrichment analysis of GO terms enriched (padj < 0.05) in DHPG/Basal RNAs (A) and RPFs (B). Gene lists were ranked by wild-type DHPG/Basal expression changes (log2FC) and p value. GO terms are separated by enrichment in either upregulated (increased) or downregulated (decreased) RNAs and RPFs. [file 13229_2020_384_MOESM1_ESM.pdf]
